# Calcified abdominal pregnancy with eighteen years of evolution: case report

**DOI:** 10.1590/S1516-31802000000600008

**Published:** 2000-11-01

**Authors:** Renato Passini, Roxana Knobel, Mary Ângela Parpinelli, Belmiro Gonçalves Pereira, Eliana Amaral, Fernanda Garanhani de Castro Surita, Caio Rogério de Araújo Lett

**Keywords:** Abdominal pregnancy, Lithopedion, Lithokelypho- pedion, Fetal death, Gravidez abdominal, Litopédio, Lithokelyphopedion, Morte fetal

## Abstract

**CONTEXT:**

The lithopedion (calcified abdominal pregnancy) is a rare phenomenon and there are less than 300 cases reported in the medical literature.

**CASE REPORT:**

In this case, a 40 year-old patient had had her only pregnancy 18 years earlier, without medical assistance since then. She came to our hospital with pain and tumoral mass of approximately 20 centimeters in diameter. Complementary examinations (abdominal X-ray, ultrasonography and computerized tomography) demonstrated an extra-uterine abdominal 31-week pregnancy with calcification areas. Exploratory laparotomy was performed, with extirpation of a well-conserved fetus with partially calcified ovular membranes.

## RESUMO

**CONTEXTO:**

O Litopédio (gestação abdominal calcificada) é um fenômeno raro, com menos de 300 casos descritos na literatura.

**RELATO DE CASO:**

Uma paciente de 40 anos, teve uma única gestação há 18 anos e não procurou serviço médico desde então. Chegou com dor em baixo ventre e massa tumoral de aproximadamente 20 centímetros. Os exames complementares (radiografia abdominal, ecografia e tomografia computadorizada) evidenciaram gestação de aproximadamente 31 semanas extrauterina e com áreas de calcificação. Foi realizada laparotomia com extração do feto em bom estado de conservação, envolvido pela membrana parcialmente calcificada.

## INTRODUCTION

Lithopedion (*litho* = stone; *pedion* = child) is the name given to an extra-uterine pregnancy that evolves to fetal death and calcification. It is a rare phenomenon that mostly comes from an abdominal pregnancy. The incidence of abdominal pregnancy is 1:11,000 pregnancies and lithopedion occurs in 1.5 to 1.8% of these cases.^1,3^ There have been less than 300 cases in 400 years of world medical literature.^2,3,4^ Because of the increase in inflammatory pelvic disease and uterine tubes surgery, there has been an increase in ectopic pregnancy.^1^ On the other hand, the occurrence of abdominal pregnancy and lithopedion has tended to become even rarer due to medical and pre-natal care becoming more accessible to the population, with the possibility of early diagnosis and treatment of the pa- thology.^1,4^

## CASE REPORT

A 40 year-old woman of brown skin had a primary complaint of lower abdomen pain. The patient reported regular abdominal growth and healthy fetal activity from a pregnancy that happened 18 years earlier. She had never done pre-natal follow-up. In the third trimester, she had started to feel strong cramps in the lower abdomen at the same time that fetal activity disappeared. She had not looked for medical assistance and some weeks later she had eliminated a dark red mass through the vagina with a placental appearance.

She had experienced the characteristic modifications of breast lactation. The abdomen had started to decrease but retained an infra-umbilical mass of about 20 centimeters in diameter, mobile and painless. A few months before being seen at our service, she started to fell pain in the lower abdomen and looked for medical assistance.

Her gynecologic history was of regular menstrual bleeding starting at the menarche and again after pregnancy. She had never used any contraceptive method.

**Figure 1:**
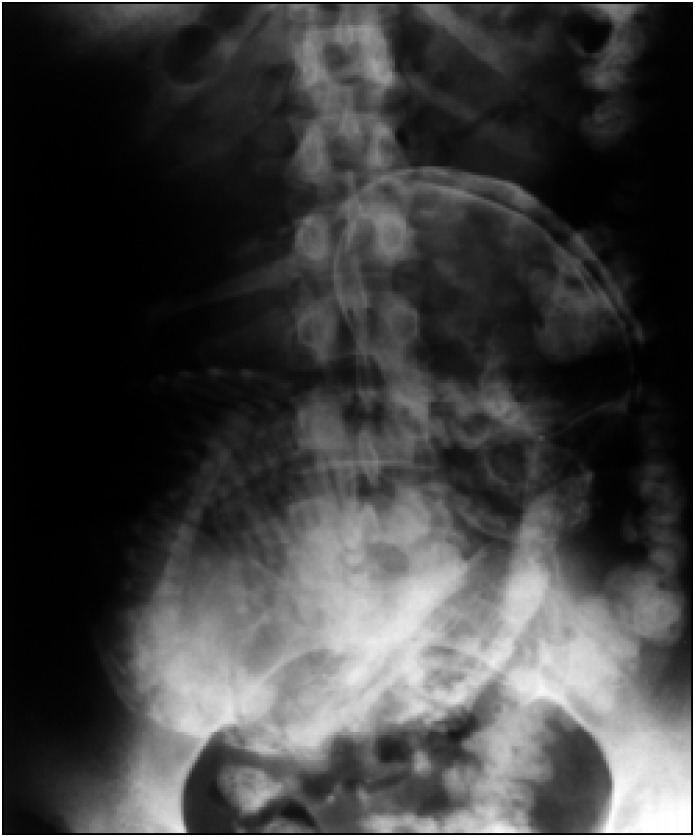
Calcified Abdominal Pregnancy - Abdominal X-ray.

The physical examination revealed an infra-umbilical mass of approximately 20 centimeters in diameter that was mobile and hardened. The uterus was without pregnancy modifications. The abdominal X-ray (Figure 1) and computerized tomography showed the presence of an ectopic fetus in a mesentery blood vessel branch, with peripheral calcifications. The ultrasound examinations showed an empty uterus, regular ovaries and the presence of a 31-week fetus (determined from femur length).

**Figure 2:**
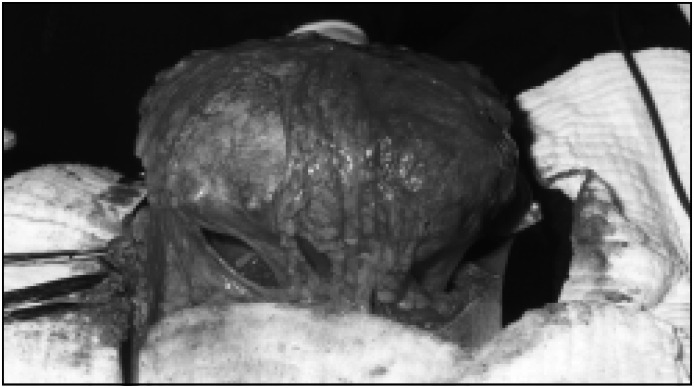
Calcified Abdominal Pregnancy - Epiploon adherence.

**Figure 3:**
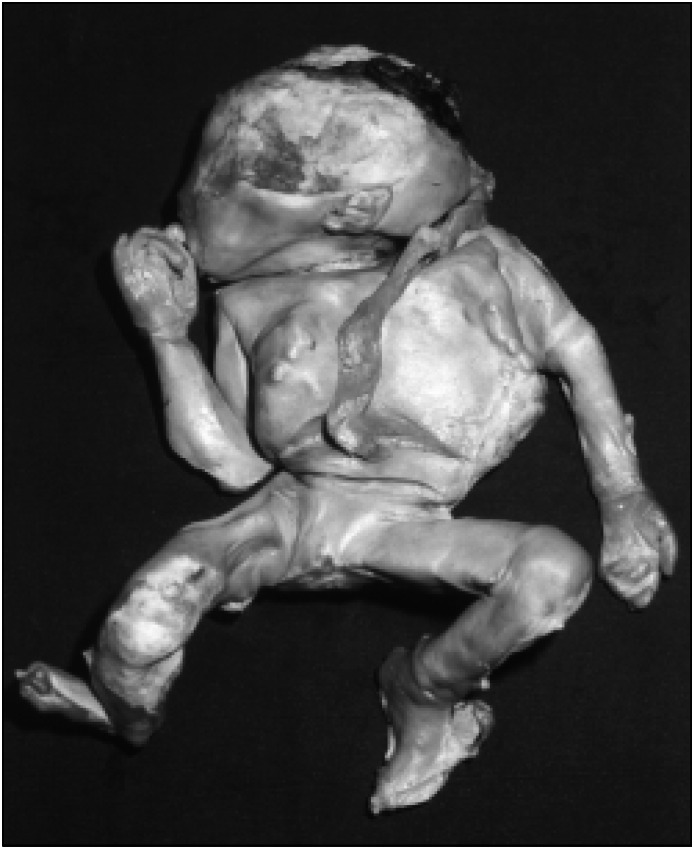
Calcified Abdominal Pregnancy - Fetus after dissection of calcified ovular membrane.

A hypothesis of lithopedion was made, and because of the clinical symptoms and the patient's desire to remove the mass, exploratory laparotomy was done. After performing parietal celiotomy, an oval tumor was seen with adherence of the right ovary and epiploon (Figure 2). It measured 15 × 25 centimeters and weighed 1,890 grams. It was composed of a calcified ovular membrane adhering to a fetus, which was dissected and proved to be well conserved and partially calcified (Figure 3). The surgery was successful, without complications, and the patient left hospital after three days.

## DISCUSSION

In the cases related in the literature, the age of the patients on the date of diagnosis varied from 23 to 100 years, 2/3 of them being over 40 years old. The period of fetus retention was from 4 to 60 years. Fetal death occurred between 3 and 6 months of pregnancy in 20% of the cases, between 7 and 8 months in 27% and at full term in 43% of the cases.^2,4^

Abdominal pregnancy results from the rupture of tubal or ovarian pregnancy with abdominal cavity implantation.^1,3^ The development of lithopedion happens under certain conditions: (1) extra-uterine pregnancy; (2) fetal death after 3 months of pregnancy; (3) the egg must be sterile; (4) there cannot be any early diagnosis; (5) local conditions must exist for calcium precipitation (deposit).^1,2,4^ The development of this pregnancy is the same as for abdominal intra-uterine pregnancy until fetal death. After this time, dehydration of tissues and calcium infiltration occur.^1,3,4^

An abdominal pregnancy that calcifies is generically called lithopedion and can have the following forms: (1) lithokelyphos (litho = rock, kelyphos = shell): only the ovular membrane is calcified and the fetus can be in different stages of decomposition; (2) lithokelyphopedion: both are calcified, i.e. fetus and ovular membrane, as in this case; (3) lithopedion: only the fetus is calcified.^4^

Although most cases remain asymptomatic for years, pelvic pain, weight sensation in the abdomen and compressive symptoms can occur, affecting especially the urinary bladder and rectum.^2,3^ Some associated complications have been reported after a long asymptomatic evolution: urinary bladder and rectum perforation; extrusion of fetal parts through the abdomen wall, rectum and vagina; intestinal obstruction (due to collision of fetal parts with the intestine or adherence) and volvulus.^3,4^

The diagnosis is revealed by a suggestive clinical history, a pelvic mass found during the physical examinations, and frequently, an X-ray of the abdomen is enough to confirm it.^3,4^ The ultrasound examination shows an empty uterine cavity and a non-specific appearance of the abdominal mass, confusing the diagnosis.^2^ Computerized tomography (CT) and nuclear magnetic resonance clearly define the pathology and help the diagnosis of adherence and other organs affected, although these are not absolutely necessary.^2,3,4^ Some authors suggest excretory urography and enema X-ray to evaluate compression or alterations in organs or systems close to it.

The diagnosis differentiates it from other calcified masses like ovarian tumors, myomas, inflammatory masses, urinary tract and bladder tumors, and epiploon calcifications.^4^ There are cases reported without surgical extirpation of the lithopedion^2^. Due to the possibility of complications, even after years of evolution, the proper procedure is surgical removal.

The surgery is frequently simple with low bleeding. No intraoperative death has been reported, even in elderly patients.^3,4^ Nevertheless, extreme care is recommended in the surgical procedure with the help of a general surgeon or urologist, due to the possibility of large quantities of abdominal blood vessels and intestinal adherence.

## References

[1] Costa SD, Presley J, Bastert G (1991). Advanced abdominal pregnancy. Obstet Gynecol Surv.

[2] Frayer CA, Hibbert ML (1999). Abdominal pregnancy in a 67-year-old woman undetected for 37 years: a case report. J Reprod Med.

[3] Irick MB, Kitsos CN, O’Leary JA (1970). Therapeutic aspects in the management of a lithopedion. Am Surg.

[4] Spiritos NM, Eisenkop SM, Mishell DR (1987). Lithokelyphos: a case report and literature review. J Reprod Med.

